# Analysis of the Properties and Thermal Behavior of Low-Temperature Phase Change Materials (PCMs) That Can Be Applied in Heating and Ventilation Systems

**DOI:** 10.3390/ma17225573

**Published:** 2024-11-14

**Authors:** Paulina Rolka, Helena Nowakowska, Marcin Lackowski

**Affiliations:** Institute of Fluid Flow Machinery, Polish Academy of Sciences, 80-231 Gdansk, Poland; helena.nowakowska@imp.gda.pl (H.N.); mala@imp.gda.pl (M.L.)

**Keywords:** phase change material (PCM), thermal energy storage (TES), latent heat thermal energy storage (LHTES), thermal characteristic, heat transfer

## Abstract

This article analyses the use of low-temperature PCMs in devices supplementing a room ventilation system to prevent the overcooling effect. In this study, the phase change is numerically simulated in an axisymmetric system consisting of two tubes. One is filled with RT11HC with an initial temperature of 0 °C, while air with an inlet temperature of 20 °C flows through the other, heating the PCM and causing it to melt. Calculations are performed using commercial software with the apparent heat method for a system of given dimensions. Spatial distributions of the system temperature and liquid volume fraction at different time moments (from 0 to 120 min) are determined. It is found that the results depended mainly on the method of determining the latent heat. For the beginning of the charging process (*t* < 40 min), the values of the liquid phase fraction determined by the H and S methods are similar, while the one determined by the G method is definitely higher (even three times at *t* = 10 min). In turn, the outlet air temperature determined by the S method is lower than that determined by the other methods. The size and shape of the mesh have no significant effect on the results.

## 1. Introduction

The increase in the concentration of greenhouse gases in the atmosphere caused by human activity brings with it unfavorable climate changes. Therefore, actions are currently being taken to carry out the energy transformation towards the decarbonization of all sectors of the economy to increase the use of renewable energy sources and reduce energy consumption. The construction sector has a significant impact on greenhouse gas emissions into the atmosphere, as energy consumption in residential and non-residential buildings amounted to 32% of the global final energy consumption (24% for households, 8% for non-residential buildings) and was responsible for 30% of CO_2_ emissions into the atmosphere [1]. As statistical data [1] indicate, the greatest energy consumption in buildings results from the need to ensure thermal comfort in them. In order to reduce energy consumption in the construction sector, the thermal modernization of buildings is carried out, consisting of replacing low energy performance heat sources, using additional insulation of buildings, replacing doors and windows with more tight ones, installing ventilation and HVAC systems, and integrating the installations of renewable energy sources with building heating and cooling systems. Although properly designed and carried out thermal modernization activities in buildings, such as increasing the building’s insulation and its tightness, may reduce the demand for heat, on the other hand, they limit the self-ventilation of rooms, which may result in exceeding the acceptable level of carbon dioxide concentration [2]. Therefore, it requires more frequent airing of rooms or the installation of special ventilation and HVAC systems. The need to ventilate rooms is particularly important in non-residential buildings and in rooms where a large number of people stay, such as office buildings, schools, offices, etc. The process of airing rooms involves heat losses. Taking the basic stock of non-industrial buildings from 13 major industrialized countries, it is estimated that the total energy demand associated with air exchange is 36% of the energy needed for the air conditioning of spaces and accounts for almost half of the losses in heating equipment [3,4]. To avoid this problem, the solution may be to use systems using phase change materials (PCMs).

PCMs, due to their ability to change their state of matter, can absorb, store or release energy in a specific temperature range. The properties of these materials allow them to be used to store energy and stabilize/maintain temperature. For this reason PCMs have found wide applications in the construction sector, where on the one hand, they are a part of thermal energy storage (TES) for storing energy, e.g., from renewable energy sources systems, and on the other hand, they are used in empty spaces of buildings to improve thermal comfort, reduce heat demand or shift the heat load from peak to off-peak periods [5,6,7,8].

Phase change materials placed in special mats, bags or panels, and located in free spaces of buildings (e.g., suspended ceilings, walls) were the subject of research in [9,10]. Lee et al. [9] used mats filled with PCM (salt hydrate with a melting point of 31 °C and a latent heat of 150 kJ/kg) attached to the walls to reduce, by approx. 27%, heat transfer on the south wall and shift the heat load by several hours. A similar effect was achieved in the tests of Berardi et al. [10], where using a commercial PCM mat (filled with BioPCM™ with a melting point of 25 °C) and a commercial panel with microencapsulated PCM (Energain panel with PCM with melting point of 21.7 °C) attached to the wall and ceiling regulated temperature fluctuations in an office building. Moreover, research results [9] showed a reduction in peak temperatures up to 6 °C.

PCMs placed in bags or special plates were also used as the accumulating material in PCM–air TESs for their integration with HVAC forced air flow systems. The connection of PCM–air TESs with HVAC systems allows for free cooling. Air (which is the heat transfer fluid (HTF) in this case) flows through the PCM–air TES and initiates heat exchange. As a result of heat exchange between the PCM and the air, the air is heated or cooled accordingly. This solution allows for many potential benefits in terms of reducing heat losses and energy consumption in the construction sector. Therefore, a lot of research has been carried out focusing on testing various designs of PCM–air TESs.

Zalba et al. [11] developed and tested a cuboidal PCM–air storage unit, inside which were rows of flat plates filled with PCM separated by empty spaces through which air flowed. In the tests, the plates used were filled with 3 kg of RT25 Rubitherm (with a phase change temperature of 19–24 °C and a latent heat of 164 kJ/kg). The test results showed that when cooling air at a temperature of 30 °C and a flow of 100 m^3^/h, the melting time of the PCM is approx. 5.5 h. However, when heating air at a temperature of 16 °C and a flow of 150 or 100 m^3^/h, the solidification time of PCM is approx. 3–4 h.

Butala and Stritih [12] investigated a PCM–air storage in the shape of a rectangular metal container with external and internal fins, filled with 3.6 kg of PCM–RT20 Rubitherm (with a melting point of 22 °C and a heat storage capacity of 172 kJ/kg), through which air was blown. Their test results show that charging this storage at night with an airflow of 15 °C allows this coolness to be used during the day and cools the air for 180–220 min at an inlet air temperature of 26 °C, for 140–150 min at an inlet air temperature of 36 °C, or for 100–110 min at an inlet air temperature of 40 °C. The flow of transferred cold ranges from 10 to 145 W.

Waqas and Kumar [13] also conducted research on a rectangular-shaped PCM–air storage with flat containers inside (filled with 13 kg of PCM–SP29 Rubitherm) to investigate the thermal performance of this unit for free cooling of buildings in dry and hot climates. Their tests focused on lowering the ambient air temperature. Waqas and Kumar’s [13] test results show that a shorter PCM solidification time (storage charging) is achieved by reducing the charging air temperature rather than by increasing the air flow rate. When the charging air temperature was reduced from 22 °C to 20 °C, it took 33% less time for the PCM to completely solidify. In the case when the air flow rate changed from 4 m^3^/h to 5 m^3^/h, the solidification time was shortened by 16%.

Arkar et al. [14] proposed a cylindrical PCM–air storage filled with PCM spheres (with RT20 Rubitherm material) through which air was passed. Their research concerned the verification of the free cooling efficiency resulting from the use of two identical PCM–air TES. The first storage was used to cool the fresh supply air and the second to cool the recirculated indoor air. The development and experimental validation of the numerical model of this storage allowed the implementation of the behavior of these units into the thermal response model of the building in the TRNSYS program. As a result, it was proven that the proposed PCM storages can reduce the size of the mechanical ventilation system.

Yang et al. [15] proposed a PCM–air TES with a cylindrical ring shape. The storage unit was filled with PCM (OP24 Ruhrtech with a melting/solidification point of 23–24 °C) and a tube was placed in the middle to ensure air flow. The proposed device was tested to verify its effect on the regulation of supply air temperature in ventilated buildings exposed to variable thermal conditions. The conducted experimental tests and numerical simulations have shown that this device enables the stabilization of air temperature fluctuations. The study shows that an average air temperature reduction of 2.5 °C was achieved, and a maximum of 5.4 °C.

Dallaire et al. [16] investigated a PCM–air TES integrated directly into the commercial available ventilation system. The storage unit has a wedge-shaped air flow configuration to provide uniform flow to two parallel PCM stacks and was designed to be attached to the back of the ventilation unit and fitted to the building ceiling. The PCM stacks consisted of 48 plates containing the SP21 EK Rubitherm material. Test results showed that the charging and discharging time of this storage unit was an average of 3.4 h, and the energy recovery efficiency of the entire system was an average of 89%.

Herbinger et al. [17] proposed a new design of a PCM–air TES system, in which the heat exchanger has a cellular structure with alternating square channels with PCM and air, so that it resembles a checkerboard pattern. The proposed PCM–air TES was to be used for hot air cooling, and its new design was expected to help improve the heat transfer rate and solve the Brate problem. Herbinger et al. [17] developed a numerical model for a fragment of the proposed storage structure. The symmetry of the numerical model included only one full and four quarter channels with PCM and four half-channels with air, in order to investigate the heat exchange process between PCM and air. In their studies, dodecanoic acid (with a melting point of 43.5 °C and latent heat of 187.2 kJ/kg) was used as the PCM. Based on the research results, it was found that the greatest influence on the heat exchange rate between the hot supply air and the PCM is exerted by the size of the channels, followed by the supply air temperature. On the other hand, the inlet velocity of the incoming air affects the total melting time of the PCM.

Cabeza et al. [18] proposed an untypical TES design that includes a new heat ex-changer resembling the vein system in plants and animals and used to increase the heat exchange surface. Cabeza et al. [18] conducted a study comparing the thermal performance of the proposed TES with a bio-inspired heat exchanger and a shell-and-tube TES. Their results indicate that the bio-inspired TES provides a shorter tank discharge time (by 52%), and the thermal parameters of the TES tank are significantly influenced by the HTF inlet temperature and, to a lesser extent, the mass flow rate.

As indicated above, the previous research work has focused mainly on proposing and testing PCM–air TES systems differing in terms of their construction (especially the type of heat exchanger) into rectangular systems containing PCM plates arranged in rows [11,12,13,16], cylindrical systems with encapsulated PCMs or PCMs placed in tubes [14,15], and bio-inspired TES [18] and cellular structures with square PCM channels [17]. The functionality tests of these solutions mainly concerned the cooling of the warm supply air; therefore, the researchers focused on checking the operating parameters, i.e., the temperature and flow rate of the supply air, the exhaust air temperature, the effect of the amount and type of PCM used, as well as the geometric properties of the PCM–air TES (e.g., thickness of the PCM plates, size of the gap through which the air flows).

In our laboratory, we are developing a low-temperature PCM-based TES designed for temperature control, especially in classrooms and office spaces during winter ventilation in cold climate regions. Such systems aim to improve energy efficiency while maintaining thermal comfort without the overcooling effect. It is assumed that the dimensions of the actual TES should not exceed 0.4 m × 0.4 m × 0.4 m, and the phase change temperature of the used PCM should be about 10 °C. The TES would be charged and discharged by forced air circulation.

Experimentally determining optimal TES parameters—such as its dimensions and HTF flow rates—is often a major challenge due to the time, materials, and financial resources required. Similarly, numerical simulations for analyzing these systems are time-consuming, and individual simulation cycles may take a long time; for example, a single computational cycle (for one set of parameters) can require up to 27 computational hours on PC with 128 GB of RAM [17]. Moreover, modeling of such systems is complicated by the uncertainties associated with the properties of the materials involved, as well as the selection of appropriate computational parameters.

The aim of this study is to evaluate how different assumptions about the thermophysical properties of PCMs affect the accuracy of TES models. In addition, our goal is to determine an optimal computational mesh that ensures that the numerical results are independent of mesh resolution while maintaining a high level of accuracy. We assumed that the system is a pipe-in-pipe structure with axial symmetry. Knowing the impact of the above factors in a simple structure can reduce the computation time for more complex, three-dimensional TESs.

Numerical calculations were performed for the TES charging process using the apparent heat capacity method and the commercial software COMSOL Multiphysics^®^ ver. 5.2 with the Microfluidics module [19,20]. This simulation software is a versatile tool for modeling and solving complex engineering and scientific problems using finite element methods [21]. It is used in fields such as fluid dynamics, heat transfer, and structural mechanics, enabling the analysis and optimization of systems and devices. The apparent heat and the fraction of the liquid phase were determined using three methods: the Heaviside function, the Gaussian function, and splines. The calculations were performed for different triangular computational meshes, differing in the size of the elements with different structures. As a result of the research, the characteristics of the temperature distribution, mass and volume fraction of the PCM liquid phase, mass flow rate of air at the outlet, and average heat flux at the outlet as a function of time were obtained.

This paper is organized as follows. In Section 2, a description of the model of the performed calculations is presented, taking into account the adopted assumptions, initial conditions, and calculation methodology. The analysis of the thermophysical properties of the low-temperature PCM (RT11 HC), as well as its apparent heat and mass fraction of liquid obtained as a result of calculations using the three methods, are described. In Section 3, the results of the heat transfer analysis and thermal behavior of RT11 HC for a single PCM-based TES section are presented and discussed. At the end of this article, the analysis results are summarized—the most important results and conclusions from the research are presented, as well as further planned research activities that allow the use of RT11 HC in a PCM-based TES dedicated for the ventilation system in practice.

## 2. Model Description

### 2.1. Assumptions

In an ideal PCM, the phase transition occurs at a strictly defined temperature (iso-thermal transition), at which there is also a sharp change in material properties, such as density or thermal conductivity [22]. In a real PCM, the phase transition does not occur at a constant temperature but over a certain temperature range. The physical properties of the material change in this interval in a smooth manner. Various methods can be used to model this behavior of real PCMs.

In this study, a method similar in principle to the one discussed in [17,22,23] was adopted. The phase transition is modeled assuming that the structure of the PCM can be approximated by two phases: solid and liquid. It is assumed that during the phase change, two phases coexist, solid and liquid, and the properties of the PCM can be determined by a single parameter denoting the mass fraction of liquid in the mixture ξ(*T*):

(1) $$\xi (T)=\frac{m^{l}}{m^{l}+m^{s}},$$

where *m^l^* and *m^s^* are, the mass of the liquid and the solid, respectively.

It is assumed that this quantity depends only on the temperature of the medium. The phase change region in which both phases exist is a homogeneous medium, and its properties depend on the properties of both phases and the local value of the parameter ξ(*T*). The dependence of all quantities on pressure is omitted.

### 2.2. Calculation Domain

**Figure 1:**
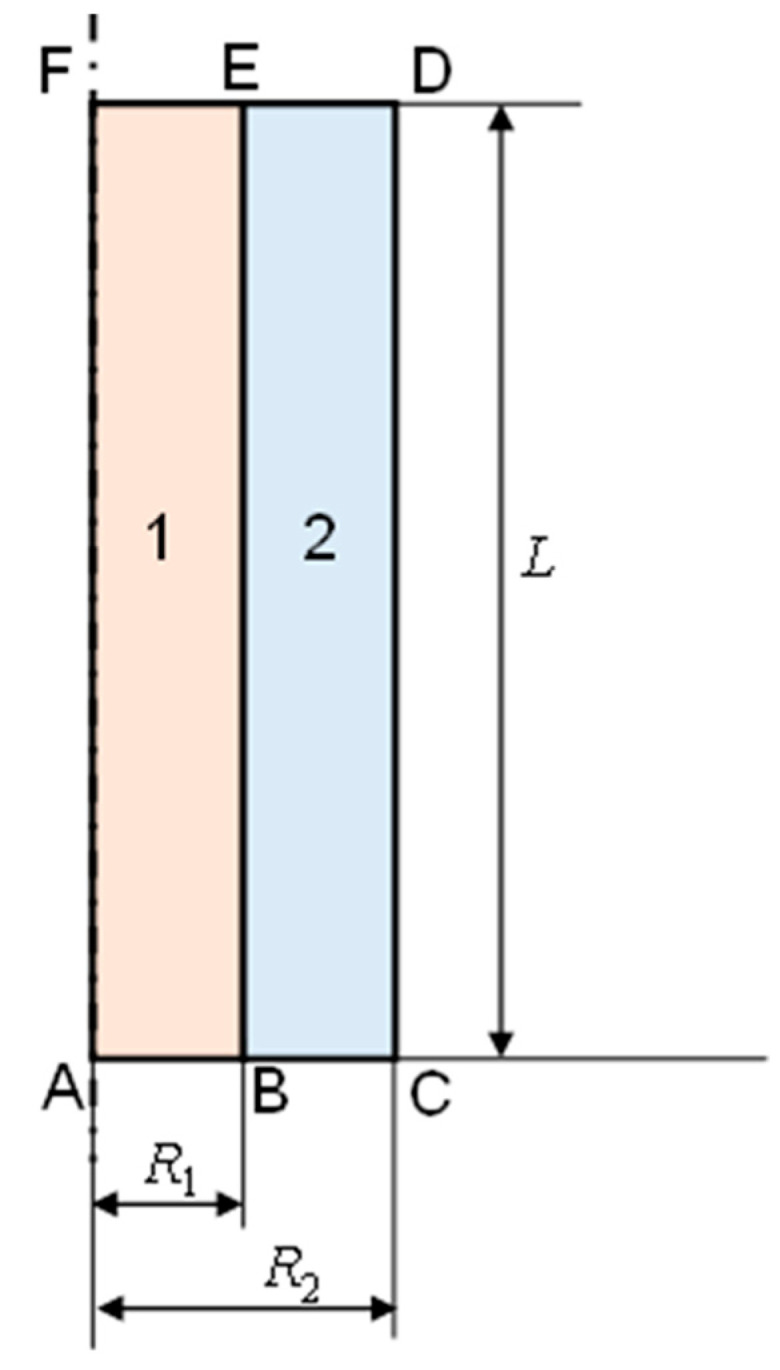
Calculation domain. Region 1 represents the PCM and region 2 represents the HTF. The A–F sections is on the symmetry axis.

As mentioned in the Introduction, the calculations were carried out assuming the axial symmetry of both the equations describing the physical phenomena and the computational domain. The domain is presented in Figure 1. It represents half of the longitudinal cross-section of the analyzed system. It consists of two regions (subdomains). Region 1 represents the PCM, while region 2 represents the HTF. The AF section is located on the axis of symmetry of the system.

### 2.3. Governing Equations

The apparent heat capacity (AHC) method was used for calculations. It was assumed that the phenomena occurring in the system can be described by energy balance equations, the form of which depends on the region. Thus, in Region 1, where the phase change occurs, when the natural convection is neglected, the energy balance is described by the equation [24]

(2) $$\rho {\tilde{C}}_{p}\frac{\partial T}{\partial t}+\nabla \cdot \left(-k\nabla T\right)=0,$$

whereas in Region 2, with HTF, by the equation

(3) $$\rho C_{p}\frac{\partial T}{\partial t}+\nabla \cdot \left(-k\nabla T\right)+\rho C_{p}u\cdot \nabla T=0.$$

In the above equations, *T* denotes temperature, ρ [kg/m^3^] denotes the density of the medium, *k* [W/(m·K)]—thermal conductivity, *C*_p_ [J/(kg·K)]—specific heat of the HTF, ***u*** [m/s]—velocity of the HTF, and ${\tilde{C}}_{p}$ [J/(kg·K)]—apparent heat capacity of the PCM, taking into account both sensible heat and latent heat. This quantity is determined in the model from the formula:

(4) $${\tilde{C}}_{p}(T)=\xi (T)C_{pl}+\left(1-\xi \left(T\right)\right)C_{ps}+C_{L}(T),$$

where *C*_pl_ is the specific heat of the substance in the liquid phase, *C*_ps_ is the specific heat of the substance in the solid phase, and the quantity *C*_L_(*T*) is the specific latent heat capacity (the distribution of the heat of phase change).

The method of determining this quantity will be discussed below. The remaining parameters of the PCM appearing in Equation (3) are determined from:

(5) $$\rho \left(T\right)=\left(1-\varphi \left(T\right)\right)\rho^{s}\left(T\right)+\varphi (T)\rho^{l}\left(T\right),$$

(6) $$k\left(T\right)=\left(1-\varphi \left(T\right)\right)k^{s}\left(T\right)+\varphi (T)k^{l}\left(T\right),$$

where ρ^s^ and ρ^l^ are the densities of the solid and liquid, respectively, *k^s^* and *k^l^* are the thermal conductivities of the solid and liquid, respectively, and φ(*T*) is the volume fraction of the liquid in the mixture and can be found from the equation [23]:

(7) $$\varphi \left(T\right)=\frac{\xi \left(T\right)}{\xi \left(T\right)+(1-\xi \left(T\right))\frac{\rho^{l}\left(T\right)}{\rho^{s}\left(T\right)}}$$

The flow of the HTF is described by the Navier–Stokes equations for an incompressible medium:

(8) ∂ρ/∂t+∇·(ρu)=0,

(9) $$\rho \;\partial u/\partial t\;+\rho \left(u\cdot \nabla \right)u=\nabla \cdot [\mu (\nabla u+{\left(\nabla u\right)}^{T}]-\nabla p,$$

where μ [kg/(m s) is the dynamic viscosity of the medium, and *p* is the pressure.

### 2.4. Methods for Determining the Distribution of the Heat of Phase Change

In this study, an analysis was carried out for three different methods of determining the values of *C*_L_ and ξ(*T*). The first method uses the Heaviside function, the second the Gaussian distribution, and the third, the spline method. The selected functions are best suitable for the numerical approximation of the thermophysical parameters of paraffins [17,23,25,26].

The method with the Heaviside function is used, among others, for modeling phase changes in the COMSOL environment [19]. In this method, it is assumed that the mass fraction of liquid in the mixture ξ(*T*) can be approximated by the function flc2hs (*T*, Δ*T*_m_/2), which is the equivalent of the smoothed and doubly differentiable Heaviside function. Its value is 0 for *T* < *T*_m_ − Δ*T*_m_/2, and 1 for *T* > *T*_m_ + Δ*T*_m_/2. The specific latent heat capacity is expressed by the equation:

(10) $$C_{L}(T)=L_{p}\frac{d\xi (T)}{dT},$$

where *L*_p_ [J/kg] is the latent heat (enthalpy of phase change).

In the Gaussian method, used, for example, in [17,25], the fraction of liquid in the mixture ξ(*T*) is approximated by a broken line:

(11) $$\xi \left(T\right)=\left\{\begin{array}{c} 0\\ \frac{T-T_{m}+\frac{\Delta T_{m}}{2}}{\Delta T_{m}}\\ 1 \end{array}\right.\begin{array}{cc} , & T<T_{m}-\frac{\Delta T_{m}}{2}\\ , & T_{m}-\frac{\Delta T}{2}<T<T_{m}+\frac{\Delta T_{m}}{2}\\ , & T<T_{m}+\frac{\Delta T_{m}}{2} \end{array},$$

while *C*_L_(*T*) is expressed by

(12) $$C_{L}(T)=L_{p}D(T)$$

and *D*(*T*) can be found from the Gaussian distribution

(13) $$D\left(T\right)=\frac{\exp\left[-{\left(T-T_{m}\right)}^{2}/{\left(\Delta T_{m}/4\right)}^{2}\right]}{\sqrt{\pi {\left(\Delta T_{m}/4\right)}^{2}}}.$$

In the spline method developed by Barz et al. [23,26], both ξ(*T*) and *C*_L_(*T*) are determined from the data provided by the manufacturers of the PCMs. These functions are represented by splines and can be determined using programs (for the MATLAB environment, among others), provided as Supplementary Materials to the article [26]. The authors’ previous studies [5,6,7] on the thermophysical properties of low- and medium-temperature commercial PCMs using DSC and T-history methods indicate that the data presented by the manufacturers are generally in good agreement with the results obtained during the experiment. Therefore, in these studies, it was decided to use the spline method based on the data provided by the manufacturers to perform the comparative analysis.

Other quantities that characterize the system’s phenomena include the mass flow rate $\dot{M}$ [kg/s] and heat flux $\dot{Q}$ [W/m^2^] of the heat transporting medium. The average values of these parameters across the outlet surface can be calculated using the following equations, respectively:

(14) $$\dot{M}=\frac{1}{\left|S\right|}\int_{S}\rho u\cdot ndS,$$

(15) $$\dot{Q}=\frac{1}{\left|S\right|}\int_{S}k\nabla T\cdot ndS,$$

where *S* is the outlet surface, |*S*| is its area, and ***n*** is a unit vector normal to the surface.

### 2.5. Materials

In the present study, it is assumed that the low-temperature PCM is paraffin with the symbol RT11HC manufactured by Rubitherm^®^ (Berlin, Germany) [27]. This material was chosen because, according to the PCM–air TES concept (described in Section 1), its phase change occurs in the temperature range of 10–12 °C, which will enable the storage of cold and the cooling of air removed from the classrooms. The properties of the RT11HC material are presented in Table 1.

It is worth noting that the thermal conductivity of the selected material (as well as other paraffins) is relatively low, which means that the heat transfer rate in a device using such a material is also relatively low (see Equation (15)). Therefore, in practical solutions, the properties of paraffins are often modified by adding particles such as graphene [8,28], graphite fibers [29,30], or metals [8]. The addition of admixtures (nanoparticles) with a high thermal conductivity coefficient increases the thermal conductivity of the base phase change material, but on the other hand, it may cause a reduction in its heat capacity, depending on the weight fraction of nanoparticles [8,28,29]. Moreover, such a measure is expensive (due to cost of admixtures), and sedimentation challenges can be expected.

**Figure 2:**
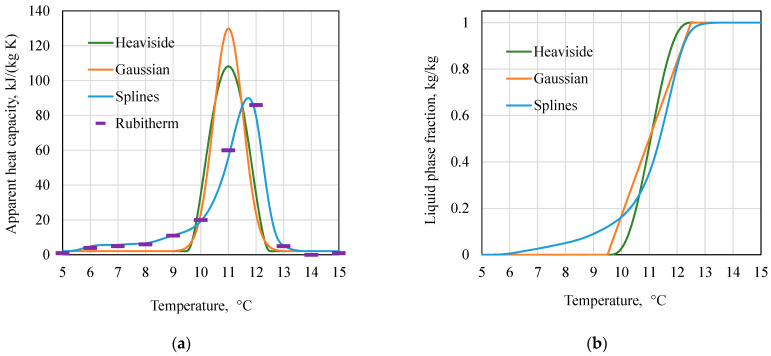
(**a**) Dependence of the apparent heat capacity ${\tilde{C}}_{p}$ on temperature determined by three methods. Additionally, data provided by the manufacturer are shown. (**b**) Dependence of the mass liquid fraction ξ on the temperature determined by three methods.

The apparent heat capacity ${\tilde{C}}_{p}(T)$ and the liquid mass fraction ξ(T) determined by three methods for RT11HC and ΔT_m_ = 3 K are shown in Figure 2.

The properties of the HTF (i.e., air) as a function of temperature were determined from the formulas provided in [31]. For selected temperatures, these values are presented in Table 2.

### 2.6. Boundary and Initial Conditions

The boundary conditions imposed for the calculations are presented in Table 3. For the calculations, it was assumed that at the initial moment, the entire system is at a constant temperature equal to *T*_0_. At the moment *t* = 0 s, the air flow begins at the inlet temperature *T*_in_. Its mass flow Φ_m_ = Φ_m,in_ in [kg/s] is a given parameter.

## 3. Results and Discussion

The calculations were performed using the COMSOL Multiphysics^®^ version 5.2 software with the Microfluidics module [20] using two interfaces: Laminar Flow and Heat Transfer in Fluids. The two interfaces were fully coupled, meaning that the fluid flow and heat transport equations were solved simultaneously. The relative tolerance adopted was 0.005; the other solver settings were default. These default settings also take into account the numerical consistent stabilization of both equations, which slightly increases the calculation time, but improves the convergence of the solution (details can be found in the software documentation [19,20]). For the assumed range of input data, no convergence problems were encountered. The following input data were assumed for the calculations: PCM—RT11HC paraffin manufactured by Rubitherm, HTF—air, inner tube radius *R*_1_ = 8 mm/√π ≅ 4.51 mm (a tube of this radius has a cross-sectional area of a square with a side of 8 mm), outer tube radius *R*_2_ = 2*R*_1_, length *L* = 30 cm, initial temperature of PCM *T*_0_ = 0 °C, air temperature at the inlet *T*_in_ = 20 °C, and mass flow rate of air at the inlet Φ_m,in_ = 1 × 10^−4^ [kg/s]. Such temperature values mean that the system is heated (charged). Moreover, one of the tubes is filled with the low-temperature PCM (RT11HC) in a solid state with an initial temperature of 0 °C, while air with an inlet temperature of 20 °C flows through the other tube, heating the PCM and causing it to melt. The calculations were performed for the three methods of determining the *C*_L_(*T*) value discussed above, i.e., using the Gaussian, Heaviside, and spline functions (marked in the figures as G, H, and S, respectively) and for three computational meshes. The choice of mesh affects the accuracy, stability, and efficiency of the resulting solution. Finer meshes improve spatial resolution and capture small-scale features and gradients but increase computational time and memory requirements. Coarser meshes reduce these overheads but can introduce errors, degrading accuracy. The optimal mesh is the one that produces reliable results at the lowest computational cost. We selected meshes with different numbers of elements, 20,000, 42,000, and 52,000 elements, and a maximum element size of 0.6 mm, 0.4 mm, and 0.4 mm, respectively. The first two meshes are uniform, the third is a non-uniform one created automatically by the software, with the mesh condensed near the wall.

**Figure 3:**
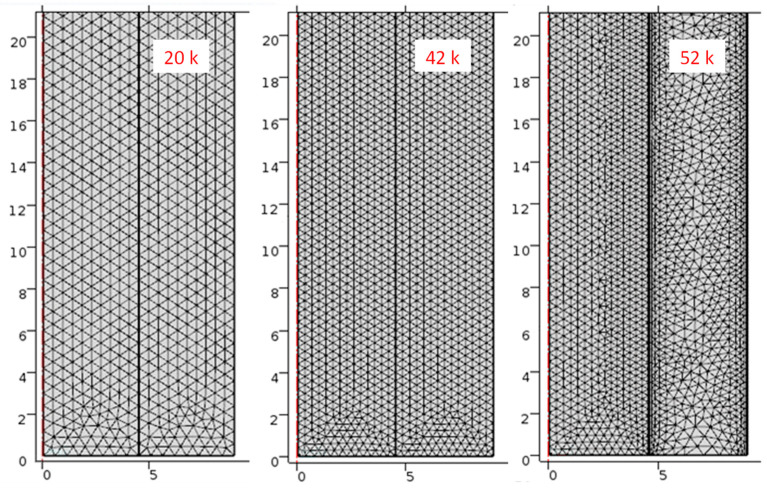
Computational meshes selected for calculations and their corresponding number of elements. Both scales are in mm.

These meshes and the corresponding numbers of elements are shown in Figure 3. We expected that the accuracy, but also the calculation time, would increase with the number of elements. In general, the calculation time depends on the configuration of the computer. For our set we computed that for a 42 k mesh, it is about two times, and for a 52 k mesh, about three times longer than for a 20 k mesh. In addition, for each of the meshes, the calculation time by method S is shorter than the other methods.

**Figure 4:**
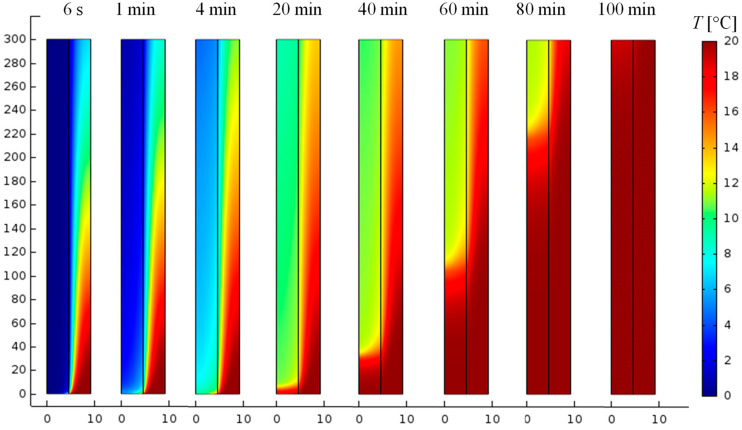
Distributions of temperature *T* in the heat storage at selected time moments (time shown at the top of the figure). Calculations for the S 20 k method. Dimensions in millimeters are given on the axes. The horizontal and vertical axes have different scales.

**Figure 5:**
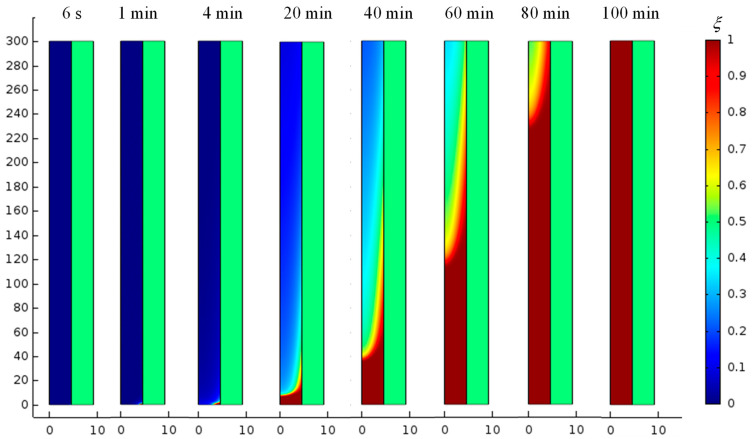
Distributions of parameter ξ for the same conditions as in Figure 4. The air-filled tube is light green.

The thermal behavior of the low-temperature PCM (RT11HC) during this study was determined by analyzing the temperature distribution of the temperature *T* and the distribution of the parameter *ξ*. This illustrated the heat exchange between the RT11HC and the flowing air. Example spatial distributions of temperature *T* and parameter ξ for selected time moments are shown in Figure 4 and Figure 5, respectively. These are the results obtained for the S 20 k method, i.e., the method in which the distribution of the phase change heat is determined by splines, for a mesh of 20 thousand elements.

**Figure 6:**
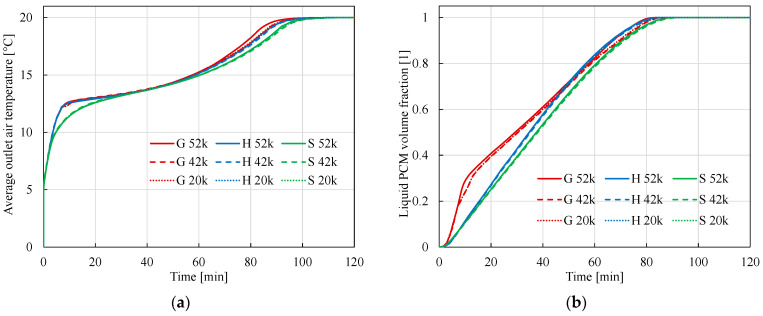
(**a**) Change over time of the average air temperature at the outlet. The symbols G, H, and S denote calculations made by the Gauss, Heaviside, and spline methods, respectively. The symbols 20 k, 42 k, and 52 k denote the approximate number of elements of the calculation mesh. Variation of average outlet air temperature over time. (**b**) Change over time of the volume fraction of liquid.

As can be seen, in the first seconds of the process, the warm air cools down as it flows through the tube. On the other hand, as a result of heat exchange between the RT11HC at the initial temperature of 0 °C and the flowing air at 20 °C, the PCM heats up and slowly melts (see Figure 4 and Figure 5). The melting of the PCM begins at the inlet and is already visible at *t* = 4 min. In the following moments of time, the temperatures of both the air and the PCM increase. It can be seen that as a result of heat exchange, the PCM reaches the phase change temperature after about 20 min, and the air temperature during this time cooled by about 2–4 K (see Figure 4). After 60 min, more than half of the PCM is in the liquid state (see Figure 5). The temperature distribution of the PCM is inhomogeneous with a rapid change in the phase transition region. This region moves in time towards the air outlet. Full melting of the material requires about 100 min (see Figure 5). After this time, the temperature of the PCM reaches 20 °C and its distribution becomes homogeneous (see Figure 4). The time dependence of the changes in the average outlet temperature is shown in Figure 6a and the changes in the volume-averaged value of the fraction ξ, in Figure 6b. During the first 10 min, the material temperature increases quickly, reaching about 12 °C; then, the rate of temperature increase is slower, and the system reaches 20 °C after about 100 min. The results obtained by the S method differ from the results obtained by the other methods: the outlet temperature is lower both in the initial and final phase of charging the TES. Between 30 and 60 min, the results obtained by the different methods practically do not differ. For the H and S methods, the choice of mesh practically does not affect the results. The maximum difference over the entire range analyzed is about 0.2 °C for both these methods. For method G, the temperature obtained for the densest mesh is the highest from approximately the 17th minute. The maximum difference obtained by this method for different meshes is 0.6 C. The maximum difference for the 52k mesh and the S method compared to the other methods is about 1.5 C.

**Figure 7:**
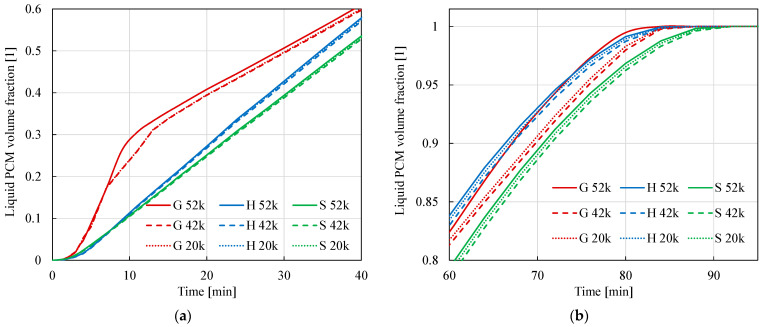
The same as in Figure 6b, only different time ranges. (**a**) the initial phase of the process (**b**) the final phase of the process.

The volume-averaged fraction ξ (i.e., the average amount of PCM melt) for methods H and S increases almost linearly with time, with the S method increasing slightly slower than for method H. For both methods, the choice of mesh has practically no effect on the results. The maximum difference over the entire analyzed range is about 0.009 for both methods. The results obtained using method G differ from the others, especially in the initial charging phase. After 40 min, they approach the results obtained using the other methods. This method is more sensitive to the choice of computational mesh. The maximum difference obtained by this method for different meshes is 0.045. The details of the results are shown in Figure 6, which shows the same results as Figure 5, but for smaller time intervals. Figure 7b shows that the full charging of the TES occurs most slowly for calculations using method S.

**Figure 8:**
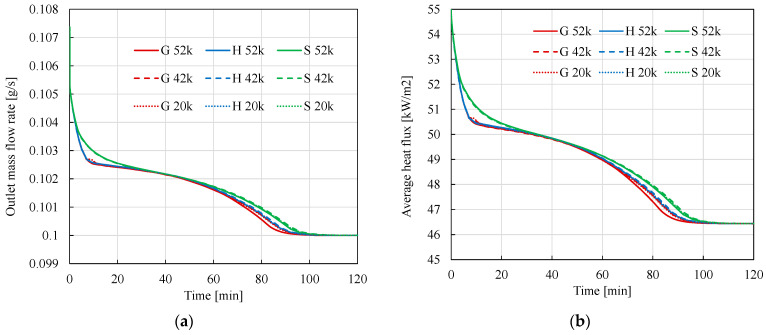
(**a**). Change over time of the mass flow rate of air at the outlet. (**b**) Change over time of the average heat flux at the outlet.

Figure 8a,b show the time evolution of the outlet air mass flow and the outlet average heat flux, respectively. These relationships are a consequence of the results shown in Figure 6a, since both of these quantities are inversely proportional to temperature. The conclusions regarding the method and meshes used are the same.

**Figure 9:**
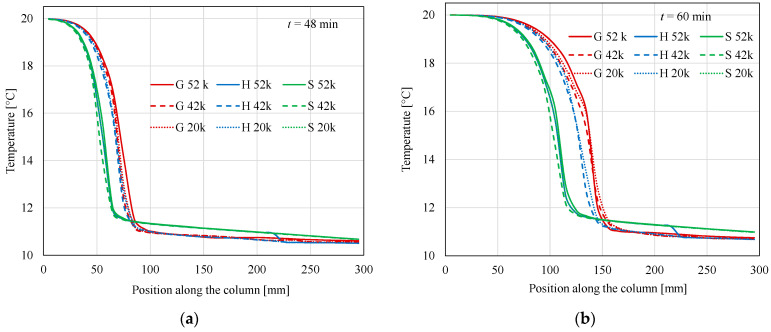

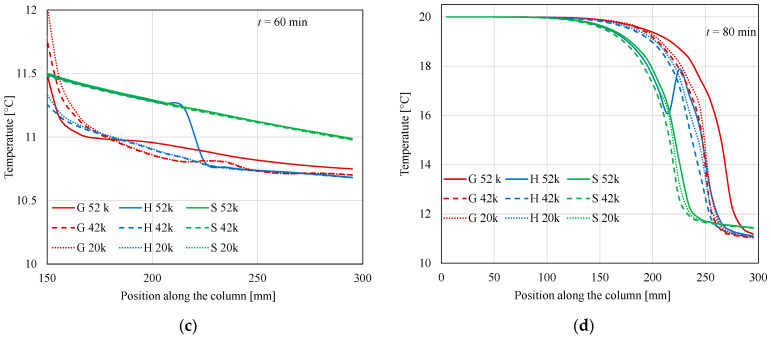
Temperature distributions on the axis for different time moments. (**a**) *t* = 48 min, (**b**) *t* = 60 min, (**c**) *t* = 60 min, position range from 150 to 300 mm, and (**d**) *t* = 80 min. Comparison of methods and meshes.

Figure 9 shows the temperature distributions of the PCM on the heat storage axis for the same methods and calculation meshes as in the previous figures, for selected time moments: *t* = 48 min, 60 min, and 80 min. As is known from the results shown earlier (Figure 7), after about 20 min, the temperature at the level of the storage inlet reaches 20 °C (i.e., air temperature at the inlet). The temperature drops to about 11 °C at the outlet. With increasing time, the melting zone moves towards the outlet. Comparing the results obtained with different methods, it can be stated that for the spline and Gauss methods, the choice of calculation mesh has no significant effect on the results. Method S gives lower temperatures than method G for smaller values of *z* and higher for larger values of *z*. Method H gives results similar to method S for *t* = 48 min and *z* < 200 mm. For larger values of *z* and the 52 K mesh, there is a “jump” to values close to those obtained by method G. This can be clearly seen in Figure 9c, where the *z* scale range has been reduced to about 50 mm. This “jump” propagates towards the outlet, as can be seen in Figure 9d. Such a jump is not visible for the results obtained with other S and G values. This is a numerical effect, which depends on the selected method and calculation mesh.

## 4. Summary and Conclusions

For modeling the process of charging the axially symmetric TES of the pipe-in-pipe type, the apparent heat capacity method was selected and the problem was solved using the commercial software COMSOL Multiphysics ver. 5.2. The apparent heat and the liquid phase fraction were determined using three methods. The quantities used were approximated by different functions: the Heaviside function (H), Gaussian function (G) and splines (S). For each function, the calculations were carried out for three triangular computational meshes, differing in the size of elements with different structures.

All methods gave basically similar results in terms of the full charging time, which is about 90 min under the assumed conditions. For the beginning of the charging process (t < 40 min), the values of the liquid phase fraction determined by the H and S methods are similar, while the one determined by the G method is definitely higher (even three times at t = 10 min). In turn, the outlet air temperature determined by the S method is lower than that determined by the other methods throughout the entire process. The G and H methods give similar results in the initial charging phase. The PCM temperature on the TES axis determined by the S method is lower than that determined by the other methods at the beginning of the system (from the inlet side), and higher in the upper part (from the outlet side). The choice of mesh did not significantly affect the results, which indicates the possibility of using the sparsest mesh, which shortens the calculation time. The calculations show that the choice of approximation for the latent heat distribution is important if accurate results are required at specific stages of the charging process. The spline method uses the apparent heat that is closest to the values provided by the manufacturer, is easy to implement in the program, and it seems that it should be used for the simulations. Confirmation of this statement would, however, require a comparison of the calculation results with experimental ones. Therefore, in the future, it is planned to perform additional experimental studies of the thermophysical properties of RT11HC to determine its heat capacity (including latent heat) and the distribution of the amount of stored energy depending on temperature using the DSC or T-history method.

This research confirms the thesis that the PCM–air TES with a low-temperature PCM (in this case, RT11HC) can be used in the future to ventilate rooms where many people are present (e.g., offices, classrooms) and contribute to reducing heat losses. The developed model will be used to perform three-dimensional calculations for a system of square-section pipes filled alternately with PCM and air. It is planned that future research will focus on analyzing the charge/discharge performance of this TES (with a cellular structure and filled with RT11HC material) intended for integration with HVAC systems.

## Figures and Tables

**Table 1:** Properties of the RT11HC material manufactured by Rubitherm^®^ [27].

Property	Value	Unit
Melting range	10–12	°C
Congealing range	12–10	°C
Heat storage capacity ± 7.5%	200	kJ/kg
Combination of latent and sensible heat in the temperature range of 5 to 20 °C	55	Wh/kg
Specific heat capacity (both phases)	2	kJ/(kg·K)
Density, solid	880	kg/m^3^
Density, liquid	770	kg/m^3^
Thermal conductivity (both phases)	0.2	W/(m·K)
Volume expansion	12.5	%
Max. operation temperature	40	°C

**Table 2:** Thermophysical properties of air.

Property	Unit	Value at 0 °C	Value at 20 °C
Density	kg/m^3^	1.293	1.205
Specific heat capacity	kJ/(kg·K)	1.0040	1.0049
Thermal conductivity	W/(m·K)	0.0242	0.0257
Dynamic viscosity	Pa·s	1.71 × 10^−5^	1.81 × 10^−5^

**Table 3:** Boundary conditions imposed for the calculations.

Boundary	Condition
AF	Symmetry
BC (inlet)	*T* = *T*_in_, Φ_m_ = Φ_m,in_
DE (outlet)	Heat flux = 0
Other external walls	Adiabatic walls
Inner walls	Temperature *T* continuity

## Data Availability

The original contributions presented in this study are included in the article. Further inquiries can be directed to the corresponding author(s).

## References

[1] Ürge-Vorsatz D., Cabeza L.F., Serrano S., Barreneche C., Petrichenko K. (2015). Heating and Cooling Energy Trends and Drivers in Buildings. Renew. Sustain. Energy Rev..

[2] Michalik M., Mizera G., Podbielska-Kubera A., Kowalski H., Cenian A. (2021). Problems with Microclimate in Classrooms—Results of Measurements and Proposed Solution. Eco-Energ. Technol. Environ. Law Econ..

[3] Liddament M.W., Orme M. (1998). Energy and Ventilation. Appl. Therm. Eng..

[4] Pérez-Lombard L., Ortiz J., Pout C. (2008). A Review on Buildings Energy Consumption Information. Energy Build..

[5] Rolka P., Przybylinski T., Kwidzinski R., Lackowski M. (2022). Thermal Properties of RT22 HC and RT28 HC Phase Change Materials Proposed to Reduce Energy Consumption in Heating and Cooling Systems. Renew. Energy.

[6] Rolka P., Kwidzinski R., Przybylinski T., Tomaszewski A. (2021). Thermal Characterization of Medium-Temperature Phase Change Materials (PCMs) for Thermal Energy Storage Using the T-History Method. Materials.

[7] Rolka P., Przybylinski T., Kwidzinski R., Lackowski M. (2021). The Heat Capacity of Low-Temperature Phase Change Materials (PCM) Applied in Thermal Energy Storage Systems. Renew. Energy.

[8] Rolka P., Przybylinski T., Kwidzinski R., Lackowski M. (2024). Investigation of Low-Temperature Phase Change Material (PCM) with Nano-Additives Improving Thermal Conductivity for Better Thermal Response of Thermal Energy Storage. Sustain. Energy Technol. Assess..

[9] Lee K.O., Medina M.A., Raith E., Sun X. (2015). Assessing the Integration of a Thin Phase Change Material (PCM) Layer in a Residential Building Wall for Heat Transfer Reduction and Management. Appl. Energy.

[10] Berardi U., Soudian S. (2019). Experimental Investigation of Latent Heat Thermal Energy Storage Using PCMs with Different Melting Temperatures for Building Retrofit. Energy Build..

[11] Zalba B., Marín J.M., Cabeza L.F., Mehling H. (2004). Free-Cooling of Buildings with Phase Change Materials. Int. J. Refrig..

[12] Butala V., Stritih U. (2009). Experimental Investigation of PCM Cold Storage. Energy Build..

[13] Waqas A., Kumar S. (2011). Thermal Performance of Latent Heat Storage for Free Cooling of Buildings in a Dry and Hot Climate: An Experimental Study. Energy Build..

[14] Arkar C., Vidrih B., Medved S. (2007). Efficiency of Free Cooling Using Latent Heat Storage Integrated into the Ventilation System of a Low Energy Building. Int. J. Refrig..

[15] Yang D., Shi R., Wei H., Du J., Wang J. (2019). Investigation of the Performance of a Cylindrical PCM-to-Air Heat Exchanger (PAHE) for Free Ventilation Cooling in Fluctuating Ambient Environments. Sustain. Cities Soc..

[16] Dallaire J., Adeel Hassan H.M., Bjernemose J.H., Rudolph Hansen M.P., Lund I., Veje C.T. (2022). Performance Analysis of a Dual-Stack Air-PCM Heat Exchanger with Novel Air Flow Configuration for Cooling Applications in Buildings. Build. Environ..

[17] Herbinger F., Bhouri M., Groulx D. (2018). Investigation of Heat Transfer inside a PCM-Air Heat Exchanger: A Numerical Parametric Study. Heat Mass Transf..

[18] Cabeza L.F., Mani Kala S., Zsembinszki G., Vérez D., Risco Amigó S., Borri E. (2024). Development of a Bio-Inspired TES Tank for Heat Transfer Enhancement in Latent Heat Thermal Energy Storage Systems. Appl. Sci..

[19] (2015). COMSOL Multiphysics Reference Manual.

[20] (2015). COMSOL Microfluidics Module User’s Guide.

[21] Zimmerman W.B.J. (2006). Multiphysics Modeling with Finite Element Methods.

[22] Barz T., Emhofer J., Marx K., Zsembinszki G. Phenomenological Modelling of Solid/Liquid Phase Transitions in Non-Ideal PCM. Proceedings of the Eurotherm Seminar #112—Advances in Thermal Energy Storage.

[23] Barz T., Bres A., Emhofer J. slPCMlib: A Modelica Library for the Prediction of Effective Thermal Material Properties of Solid/Liquid Phase Change Materials (PCM). Proceedings of the Asian Modelica Conference.

[24] Prakash S.A., Hariharan C., Arivazhagan R., Sheeja R., Raj V.A.A., Velraj R. (2021). Review on Numerical Algorithms for Melting and Solidification Studies and Their Implementation in General Purpose Computational Fluid Dynamic Software. J. Energy Storage.

[25] Herbinger F., Bhouri M., Groulx D. (2016). Numerical Study of a PCM-Air Heat Exchanger’s Thermal Performance. J. Phys. Conf. Ser..

[26] Barz T., Krämer J., Emhofer J. (2020). Identification of Phase Fraction–Temperature Curves from Heat Capacity Data for Numerical Modeling of Heat Transfer in Commercial Paraffin Waxes. Energies.

[27] Rubitherm Data Sheet RT11HC. https://www.rubitherm.com/media/products/datasheets/Techdata_-RT11HC_EN_08102020.PDF.

[28] Rolka P., Lackowski M. (2023). The Effect of Graphene Nanoparticleson the Thermal Conductivity Enhancementof Organic Phase Change Material and Its Energystorage Properties. Arch. Thermodyn..

[29] Wang L., Liu Z., Guo Q., Wang H., Wang X., Dong X., Tian X., Guo X. (2022). Preparation and Thermal Characterization of Hollow Graphite Fibers/Paraffin Composite Phase Change Material. Coatings.

[30] Wang L., Liu Z., Guo Q., Yang J., Dong X., Li D., Liu J., Shi J., Lu C., Liu L. (2015). Structure of Silicon-Modified Mesophase Pitch-Based Graphite Fibers. Carbon.

[31] Dixon J.C. (2007). Appendix B: Properties of Air. The Shock Absorber Handbook.

